# Supplementary material for: Splice-Junction-Based Mapping of Alternative Isoforms in the Human Proteome
Source: Cell Rep. Author manuscript; Available in PMC 2020 Jan 15. (PMC6961840; doi:10.1016/j.celrep.2019.11.026)
Supplement: 2 [file NIHMS1546469-supplement-2.zip › DF1/PXD006675/RightCoronaryArtery/RightCoronaryArtery_11_RNF14_CPSVATPGQRNEDELLALASIYDGDEFR.pdf]

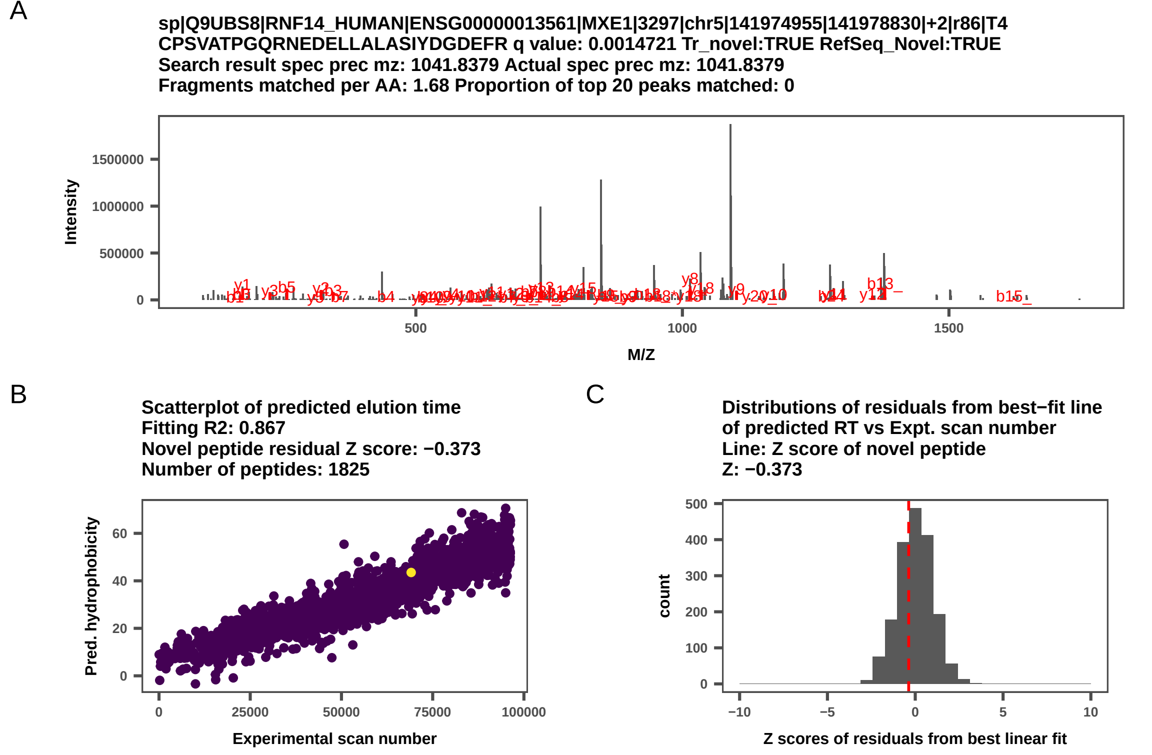
A
sp|Q9UBS8|RNF14_HUMAN|ENSG00000013561|MXE1|3297|chr5|141974955|141978830|+2|r86|T4
CPSVATPGQRNEDELLALASIYDGDEFR q value: 0.0014721 Tr_novel:TRUE RefSeq_Novel:TRUE
Search result spec prec mz: 1041.8379 Actual spec prec mz: 1041.8379
Fragments matched per AA: 1.68 Proportion of top 20 peaks matched: 0
Intensity
1500000
1000000
500000
0
500
1000
1500
M/Z
B
Scatterplot of predicted elution time
Fitting R2: 0.867
Novel peptide residual Z score: −0.373
Number of peptides: 1825
Pred. hydrophobicity
60
40
20
0
0
25000
50000
75000
100000
Experimental scan number
C
Distributions of residuals from best−fit line
of predicted RT vs Expt. scan number
Line: Z score of novel peptide
Z: −0.373
count
500
400
300
200
100
0
−10
−5
0
5
10
Z scores of residuals from best linear fit
